# Italian Biodiversity: A Source of Edible Plant Extracts with Protective Effects Against Advanced Glycation End Product-Related Diseases

**DOI:** 10.3390/nu17060935

**Published:** 2025-03-07

**Authors:** Giulia Moretto, Raffaella Colombo, Stefano Negri, Hellas Cena, Lorena Vailati, Adele Papetti

**Affiliations:** 1 Drug Sciences Department, University of Pavia, 27100 Pavia, Italy; giulia.moretto01@universitadipavia.it (G.M.); raffaella.colombo@unipv.it (R.C.); 2 National Biodiversity Future Center (NBFC), 90133 Palermo, Italy; stefano.negri@univr.it (S.N.); hellas.cena@unipv.it (H.C.); 3 Department of Biotechnology, University of Verona, 37134 Verona, Italy; 4 Department of Public Health, Experimental and Forensic Medicine, University of Pavia, 27100 Pavia, Italy; 5 Clinical Nutrition Unit, ICS Maugeri, Istituti di Ricovero e Cura a Carattere Scientifico (IRCCS), 27100 Pavia, Italy; 6 Struttura Complessa di Anatomia Patologica, Fondazione Istituto di Ricovero e Cura a Carattere Scientifico (IRCCS) Policlinico San Matteo, 27100 Pavia, Italy; l.vailati@smatteo.pv.it

**Keywords:** biodiversity, edible plant extract, AGE-related diseases, antiglycative agents, hypoglycemic effect, amyloid beta peptide

## Abstract

**Background**: Italy’s plant biodiversity, characterized by many plant species, is an important source of bioactive secondary metabolites that help reduce the risk of the development of advanced glycation end product (AGE)-related diseases. AGEs are involved in various diseases, such as diabetes, cardiovascular, and neurodegenerative disorders. Therefore, the aim of the study was to investigate the antiglycative, hypoglycemic, and neuroprotective properties of nine edible plant extracts using different in vitro assays. **Methods**: The ability of the extracts to counteract AGE formation was evaluated at different stages of the glycation reaction using in vitro systems based on the determination of Amadori products and the co-incubation of a model protein with a dicarbonyl compound under different experimental conditions. In addition, the extracts’ methylglyoxal (MGO) and glyoxal (GO) trapping ability was investigated. Hypoglycemic activity was assessed by measuring α-amylase inhibition, while the neuroprotective effects were explored by testing amyloid β peptide 1-42 (Aβ1-42) fibrillogenesis inhibition. **Results**: All extracts generally had a dose-related capacity for the inhibition of AGE formation, mainly at the intermediate stage of the glycation reaction; high trapping capacity against MGO and GO; and promising hypoglycemic properties. In addition, they affected the fibrillogenesis process by reducing mature amyloid fibril formation and altering fibril morphology. **Conclusions**: All tested extracts had promising anti-fibrillogenic properties. *Rosa canina* extract was the most active among the tested plant species given its antiglycative activity (about 80% inhibition of AGE formation), trapping capacity against MGO and GO (almost 100%), hypoglycemic effects (66.20 ± 0.88%), and anti-fibrillogenic effects (69.00 ± 4.49% inhibition), indicating its suitability in the management of AGE-related diseases and for the potential development of a novel food ingredient.

## 1. Introduction

Following the “One Health” approach established by the World Health Organization [1] in the last decade, many researchers have focused attention on investigating how the health of animals, humans, and ecosystems could be interconnected and related to each other. In this framework, one topic was to investigate how biodiversity could affect human health [2,3]. The negative effects of anthropism and climate change on biodiversity are evident with local and global extinctions. Therefore, preserving and enhancing it as a source of goods and resources is important to maintain healthy ecosystems and coexistence between humans and nature, which will be sustainable in the long term and resilient to ongoing climate change. Italy has one of the most significant biodiversity heritages in Europe, both in terms of the total number of plants and the high rate of endemism. In particular, the flora consists of a high number of species and subspecies due to the lithological, climatic, and topographical diversity of the territory. Natural compounds have been used as drugs since ancient times. Scientific advances over time have enabled the identification of biologically active compounds, commonly referred to as secondary metabolites, to which various biological properties are attributed. In recent years, the use of natural plant extracts as food supplement ingredients has increased due to their healthy properties, which are useful in the risk reduction of the development of many diseases [4,5]. In particular, several studies indicated how plants and their metabolites play a role in the prevention and treatment of chronic degenerative diseases, such as diabetes [6] and neurodegenerative disorders, including Alzheimer’s disease (AD) [7]. These chronic disorders are strictly correlated to the formation of advanced glycation end products (AGEs) involved in aging processes. In fact, the high formation of AGEs is a consequence of hyperglycemia, which causes diabetes-related complications (microvascular and macrovascular disorders) and contributes to amyloidosis in AD [8,9]. In addition, glycation can modulate the self-assembly and fibrillogenesis of amyloid β (Aβ) peptides, leading to the formation of AGE–Aβ peptides, which are more toxic and able to accelerate AD progression [9,10].

Considering the above mentioned, the aim of the present research was to investigate the antiglycative properties of *Althaea officinalis* L. (Malvaceae family), *Arthemisia abrotanum* L. (Asteraceae family), *Salvia pratensis* L. (Lamiaceae family) *Castanea sativa* L., (Fagaceae family), *Verbaschum thapsus* L. (Scrophulariaceae family), *Beta vulgaris* L. (Amaranthaceae family), *Butomus umbellatus* L. (Butomaceae family), *Sanguisorba officinalis* L., and *Rosa canina* L. (Rosaceae family) polyphenolic extracts. In the literature, many bioactivities have been ascribed to these species, such as antibacterial, antiviral, antifungal, antioxidant, anti-inflammatory, and antiproliferative effects [11,12,13,14,15,16,17]. Conversely, no investigations about antiglycative properties can be found. Therefore, we evaluated the above mentioned extracts’ capacity to interfere with AGE formation in the initial or intermediate step of the glycation reaction, glucose levels, and the amyloid β peptide 1-42 (Aβ1-42) fibrillogenesis process. In addition, considering that dicarbonyl compounds (methylglyoxal-MGO and glyoxal-GO) can be generated during the glycation reaction [18,19], the extracts’ capacity to directly trap MGO and GO was monitored.

The obtained in vitro results could help to select some extracts potentially useful as promising antiglycative agents that obviously need further investigation regarding their chemical characterization, bioaccessibility, stability, safety, and in vivo testing before they are marketed.

## 2. Materials and Methods

### 2.1. Experimental Approach

In the framework of the National Biodiversity Future Center (NBFC) research project (funded by the MUR through European Union funds—NextGenerationEU), nine edible plant species according to the Belfrit List (*Althaea officinalis* L. (Malvaceae family), *Arthemisia abrotanum* L. (Asteraceae family), *Salvia pratensis* L. (Lamiaceae family), *Castanea sativa* L. (Fagaceae family), *Verbaschum thapsus* L. (Scrophulariaceae family), *Beta vulgaris* L. (Amaranthaceae family), *Butomus umbellatus* L. (Butomaceae family), *Sanguisorba officinalis* L., and *Rosa canina* L. (Rosaceae family)) were selected for the investigation (Table 1). A literature research about their bioactivities was performed using Scopus, PubMed, and Google Scholar databases. Considering that no antiglycative properties are reported, we focused on such properties, and the most used in vitro and enzymatic assays were selected for this first part of this research project.

### 2.2. Plant Material

Fresh plant material was collected from vegetative aerial organs and ground in liquid nitrogen using an A11 basic analytical mill (IKA-Werke, Staufen, Germany). The sampling features of each plant are reported in Table 1. The resulting frozen powders were extracted with 10 volumes (1 g/10 mL) of LC-MS grade methanol. The samples were vortexed for 30 s, sonicated for 10 min at 40 kHz in an ultrasonic bath (SOLTEC, Milan, Italy) with ice, and centrifuged at 14,000× *g* for 10 min at 4 °C. Supernatants were split into 1 mL aliquots, each derived from 100 mg of fresh plant material, dried using a Speed-Vac system (He-to-Holten, Frederiksborg, Denmark), and stored at −20 °C. Each aliquot was then differently dissolved and diluted according to the assay.

### 2.3. Reagents

HPLC grade acetic acid and methanol; 1,1,1,3,3,3-hexafluoro-2-propanol (HFIP, purity grade ≥ 99%); type VI-B porcine pancreatic α-amylase (≥5 units/mg solid); dinitrosalicylic acid (DNS, purity grade ≥ 98%); methylglyoxal (MGO, 40% aqueous solution); glyoxal (GO, 40% aqueous solution); aminoguanidine hydrochloride (AG, purity grade ≥ 98%); nitrotetrazolium blue chloride (NBT, purity grade ≥ 90%); bovine serum albumin (BSA, purity grade ≥ 98%); D-(+)-glucose (GLU, purity grade ≥ 99.5%); 5-methylquinoxaline (5-MQ, purity grade ≥ 98%); *o*-phenylenediamine (OPD, purity grade ≥ 98%); sodium dihydrogen phosphate monohydrate (purity grade ≥ 98%); disodium hydrogen phosphate dodecahydrate (purity grade ≥ 99%); and sodium azide (purity grade ≥ 99.5%) were provided by Merck Life Science S.r.l. (Milan, Italy). Sodium potassium tartrate tetrahydrate (purity grade ≥ 99%), sodium hydroxide pellets (purity grade ≥ 97%), sodium chloride (purity grade ≥ 99.5 %), ethanol (96%), sodium bicarbonate (purity grade ≥ 99.7%), and sodium carbonate anhydrous (purity grade ≥ 99.7%) were supplied by Carlo Erba (Milan, Italy). LC-MS grade methanol was provided by Honeywell (Seelze, Germany). Thioflavin T (ThT) was provided by Thermo Fisher Scientific (Waltham, MA, USA). Synthetic Aβ1-42 (MW 4514.10 Da, purity grade ≥ 95%) was purchased as freeze-dried powder from Anaspec (Fremont, CA, USA). Carbon-coated formvar nickel grid (200 mesh) and uranyl acetate were purchased from Electron Microscopy Sciences (Washington, PA, USA). Water was obtained from a Millipore Direct-QTM system (Merck-Millipore, Milan, Italy).

### 2.4. Evaluation of Amadori Product Inhibition

The Amadori products were measured as fructosamine with NBT salt according to the assay described by Zhang et al. [20] with slight modifications [21,22]. Each sample was prepared by dissolving the dry material in a 2 mL aliquot of the EtOH:H_2_O mixture at 1:3 *v*/*v* and then diluting it with phosphate buffer (100 mM, pH 7.4, 0.02% NaN_3_) to obtain 0.5 and 1 mg/mL final concentrations in the reaction mixtures. In the presence of fructosamine, NBT is reduced to the tetrazinolyl radical, forming monoformazano (MF+), a colored compound spectrophotometrically measured at 530 nm (Spectrophotometer Lambda 25, Perkin Elmer, Milan, Italy). The capacity of extracts to inhibit fructosamine formation was calculated as reported by Maietta et al. [22].

### 2.5. Evaluation of MGO and GO Trapping Capacity Using the RP-UHPLC-DAD Method

Direct GO and MGO trapping ability was determined as described by Mesías et al. [23] and Maietta et al. [21,22] with slight modifications. GO or MGO was incubated for 24, 48, 72, and 96 h at 37 °C in the presence or absence of the extract sample solution. Extract sample solutions were prepared by dissolving each dry material in an EtOH:H_2_O mixture at 1:3 *v*/*v* and then diluting it with phosphate buffer (100 mM, pH 7.4, 0.02% NaN_3_) to obtain 0.5 and 1 mg/mL final concentrations in the reaction mixtures. The residual GO and MGO were quantified as quinoxaline derivatives using the RP-UHPLC-DAD method. A Vanquish UHPLC system (Thermo Fisher Scientific, Waltham, MA, USA) equipped with an autosampler, binary pump, column compartment, and diode array detector (DAD) was used for the analyses. The separation was carried out on a Gemini^®^ NX-C18 analytical column (150 × 2.0 mm i.d., 3 μm, Phenomenex, Torrance, CA, USA) in isocratic mode using a mobile phase consisting of 0.5% acetic acid aqueous solution and methanol at 50/50, *v*/*v* and a constant flow rate of 0.4 mL/min. The injection volume was 20 μL. The column temperature was set at 40 °C, and 5-MQ was used as the internal standard. Chromatograms were recorded at 315 nm. Trapped GO and MGO were calculated as reported by Maietta et al. [22].

### 2.6. Evaluation of the Extracts’ Capacity to Inhibit AGE Formation

The antiglycative properties of all extracts at the intermediate step of the glycation reaction were determined using the BSA-MGO assay [21,22,23]. A model system consisting of BSA and MGO was incubated for 1, 4, and 7 days at 37 °C in the presence or absence of the extract solution. The extracts were prepared by dissolving each dry material in an EtOH:H_2_O mixture at 1:3 *v*/*v* and then diluted with phosphate buffer (100 mM, pH 7.4, 0.02% NaN_3_) to obtain 0.5 and 1 mg/mL final concentrations in the reaction mixtures. Here, 0.5 mg/mL AG was used as a positive control. The fluorescence intensity (FI) of argpyrimidine-like (λ_exc_ 335 nm; λ_em_ 440 nm) and vesperlysine-like (λ_exc_ 370 nm; λ_em_ 440 nm) AGEs was monitored (Spectrofluorometer L550B, Perkin Elmer, Milan, Italy), and the capacity of each extract sample to inhibit AGE formation was calculated, as reported by Maietta et al. [22].

### 2.7. Evaluation of the Extracts’ Capacity to Inhibit α-Amylase Activity

The hypoglycemic property of extracts was evaluated using an in vitro α-amylase enzymatic assay, as described by Ferron et al. [24]. Sample extract solutions were prepared by dissolving each dry material aliquot in EtOH:H_2_O at 1:3 *v*/*v* and then diluting them with phosphate buffer (100 mM, pH 7.4) to obtain 0.1 mg/mL, 0.25 mg/mL, 0.5 mg/mL, and 1 mg/mL final concentrations in the reaction mixture. The capacity of extracts to inhibit α-amylase activity was calculated as reported by Ferron et al. [24].

### 2.8. Thioflavin T Fluorescence Assay

The ThT fluorescence assay was performed to monitor the in vitro fibrillogenesis process of Aβ1-42 in the presence or absence of the extracts.

Aβ1-42 was reconstituted in HFIP to a final concentration of 0.5 mg/mL, gently mixed, and stored for 30 min at 4 °C to promote dissolution. The obtained peptide solution was aliquoted in microfuge tubes, and HFIP was evaporated using a Speed-Vac (Concentrator plus, Eppendorf, Hamburg, Germany). Each resulting peptide film (containing 100 μg of peptide) was stored at −80 °C until used [25].

ThT assay solution was prepared by diluting 200 μM ThT stock solution (in 0.02 M phosphate buffer, pH 7.4) to obtain a 1.5 μM final concentration. Then, in a 96-well black-bottomed microplate, 50 μM Aβ1-42 solution was incubated with ThT (1.5 μM), with or without 100 μL/well of extract (1 mg/mL phosphate buffer). The microplate was placed in a microplate reader (FLUOstar omega, BMG Labtech, Ortenberg, Germany) thermostatted at 37 °C to promote and monitor the fibrillogenesis process for 72 h. The fluorescence of amyloid fibril-bound ThT was measured every 15 min (λ_exc_ 448 nm; λ_em_ 482 nm). The microplate was shaken (200 rpm) for 300 s after each measurement to accelerate fibril formation. Sample fluorescence was recorded after subtraction of free ThT fluorescence. In addition, the absence of the intrinsic fluorescence of each extract was verified. Resveratrol, a well-known fibrillogenesis process inhibitor, was used as a positive control (100 μg/mL) [26]. Three independent experiments were performed, and tests were run in triplicate in each experiment. The percentage of inhibition of Aβ1-42 fibril formation was calculated using the following formula:

$$\mathrm{Inhibition}\;(\%)=\left(\frac{F_{0}-F_{1}}{F_{0}}\right)\times 100$$

where *F*_0_ and *F*_1_ are the highest relative fluorescence of Aβ1-42 peptide and the relative fluorescence of Aβ1-42 incubated with extract, respectively.

### 2.9. Transmission Electron Microscopy (TEM)

Ten microliters of each sample were applied to a carbon-coated formvar nickel grid. The samples were sedimented on the carbon film for 15 min and then negatively stained with 10 μL of 2% uranyl acetate, *w*/*v*. After draining off the excess of staining solution, the specimen was transferred to the electron microscope (JEM 1400 Plus, JEOL Ltd., Tokyo, Japan), operating at 80 kV. An AMT V700 camera was used to take photos of electron micrographs of negatively stained samples.

### 2.10. Statistical Analysis

The data are the mean ± standard deviation (SD) of three independent experiments, each performed in triplicate (n = 9).

Statistical analysis of the data was performed using Microsoft Office 365. The significant differences were evaluated using two-way ANOVA followed by Tukey post hoc test (*p* < 0.05 or *p* < 0.01).

Principal component analysis (PCA) was performed using the R-based software Chemometric Agile Tool (version 3.0.0) to summarize the data obtained on the antiglycative and hypoglycemic properties of the tested extracts. All data sets were mean centered and scaled to allow all variables to contribute equally to the model. In addition, linearity and multicollinearity were assessed by calculating the correlation coefficients and correlation matrix, respectively.

## 3. Results and Discussion

Considering the growing interest in the literature about the capacity of plant extracts rich in polyphenols to act at different stages of the glycation reaction [27], suitable in vitro assays were selected to investigate the ability of the tested extracts to interfere with the glycation process. In particular, the initial stage of the reaction was studied using the BSA-NBT assay to monitor the formation of the Amadori products. In fact, the first product of the nonenzymatic glycation (involving the primary amine groups, mainly of arginine, lysine, or protein N-terminus) is an unstable imine derivative (Schiff’s base) that undergoes rearrangement and generates the Amadori products, important precursors of several reactive α-dicarbonyl intermediates, such as MGO and GO, which have a critical relevance in the formation of AGEs [28]. Therefore, the ability of the extracts to inhibit the formation of Amadori products was tested at 0.5 mg/mL and 1 mg/mL. Such compounds have been generated in the model system following the incubation of glucose (used as a glycative agent) and BSA (used as a model protein) for 14 days according to the literature since the kinetics of their formation is slow as a result of the different Schiff’s base rearrangements [29]. Overall, the extracts were unable to reduce the formation of the Amadori products, and only *A. officinalis*, *S. pratensis*, *R. canina*, and *B. umbellatus* had activity values lower than 15.00 ± 3.00% when tested at the highest concentration.

The intermediate stage of the glycation reaction, which is characterized by the condensation of α-dicarbonyl compounds (generated by enolization processes of the Amadori products under different conditions or by glycoxidation) and the amino groups of lysine/arginine and the subsequent formation of AGEs [28], was investigated using the BSA-MGO model system. The ability of the extracts (0.5 and 1 mg/mL) to inhibit the formation of argpyrimidine-like (generated by the interaction between the MGO and the guanidine group of an arginine residue) and vesperlysine-like (resulting from glycoxidation processes) fluorescent AGEs was monitored after 1, 4, and 7 days of incubation, according to MGO glycation kinetics. A dramatic reduction in AGE formation is observed after 7 days of incubation [22,28,29,30,31,32]. AG was always used as positive control due to its well-known antiglycative properties. In fact, AG reduced the formation of argpyrimidine-like and vesperlysine-like AGEs by 90% and 70%, respectively, at each incubation time.

**Figure 1:**
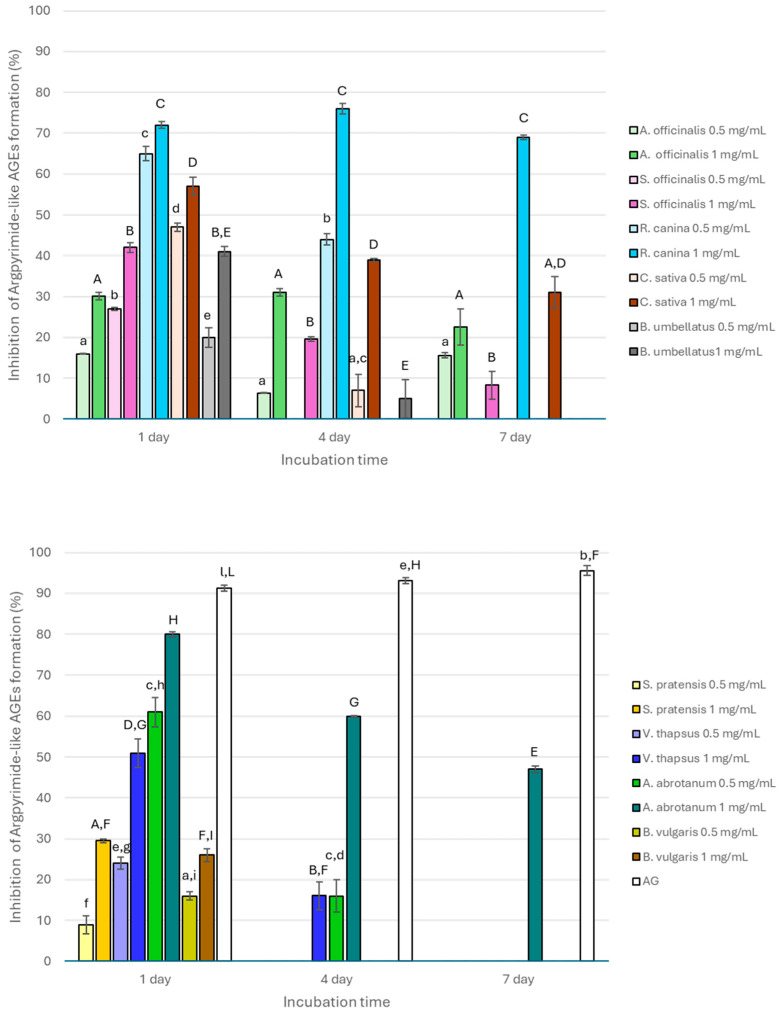
Antyglycative activity of the tested extracts (0.5 and 1 mg/mL) and AG on the formation of argpyrimidine-like AGEs. Different superscript letters (lowercase and capital letters refer to 0.5 mg/mL and 1 mg/mL, respectively) within each monitoring time indicate significant differences (*p* < 0.05) among extracts and AG.

All extracts inhibited the formation of argpyrimidine-like AGEs after 1 day of incubation (Figure 1) with values ranging from 8.98 to 80.00% and showing a dose-dependent antiglycative property. The activity generally decreased after 4 days of incubation and completely disappeared after 7 days, with the exception of *S. officinalis*, *R. canina*, *C. sativa*, and *A. abrotanum*, which were still active only at 1 mg/mL (inhibitory values of 8.33 ± 3.42%, 69.00 ± 0.49%, 31.00 ± 3.92%, and 46.98 ± 0.48%, respectively). In contrast, *A. officinalis* was still active at both the tested concentrations (15.58 ± 0.64% and 22.56 ± 4.39% at 0.5 mg/mL and 1 mg/mL, respectively). The activity registered for all the extracts was always significantly (*p* < 0.05) lower than that registered for AG, which had inhibitory values higher than 90%. *R. canina*, *C. sativa*, and *A. abrotanum* were the most active, and their activity significantly decreased (*p* < 0.01) with the increase in monitoring time, except for *R. canina*, which had a particular trend due to the significant increase in its activity after 4 days followed by a significant decrease at the end of the incubation time (*p* < 0.01) when tested at 1 mg/mL.

**Figure 2:**
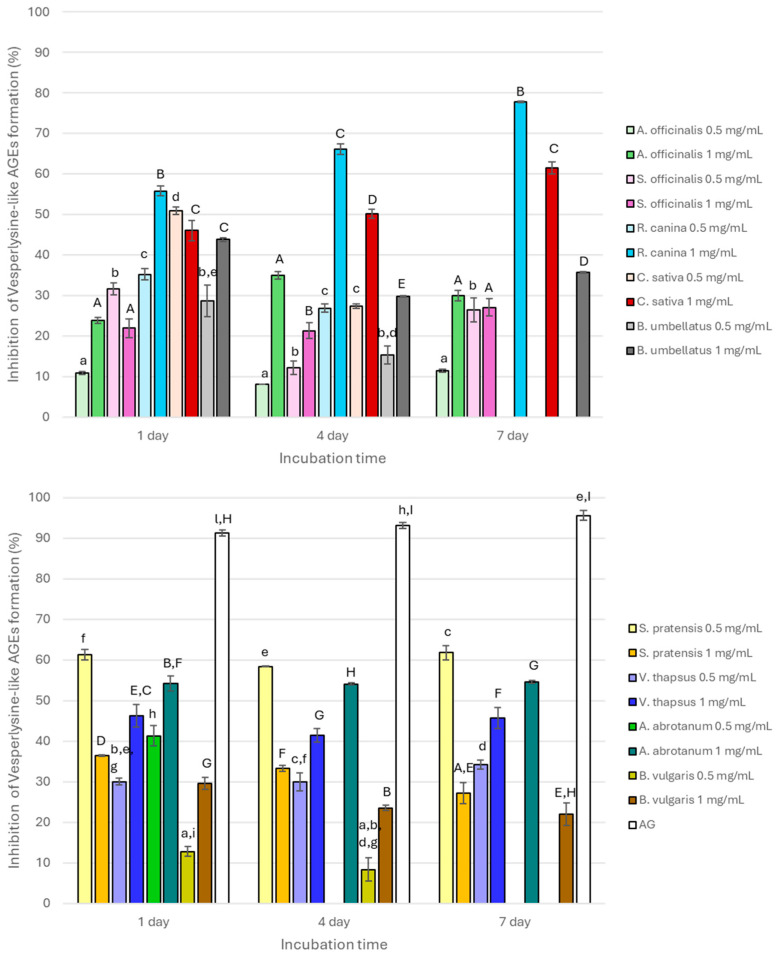
Antyglycative activity of the tested extracts (0.5 and 1 mg/mL) and AG on the formation of vesperlysine-like AGEs. Different superscript letters (lowercase and capital letters refer to 0.5 mg/mL and 1 mg/mL, respectively) within each monitoring time indicate significant differences (*p* < 0.05) among extracts and AG.

In addition, all the extracts had also high capacity to inhibit vesperlysine-like AGE formation. However, in contrast to that mentioned above for argpyrimidine-like AGEs, a dose-dependent activity was not always observed (Figure 2). In fact, *S. officinalis* and *C. sativa* had significantly higher activity when tested at 0.5 mg/mL (*p* < 0.01) at the beginning of the monitoring period (after 1 day of incubation). Then, for longer incubation periods, a dose-dependent inhibitory capacity was registered with values significantly higher both after 4 and 7 days for 1 mg/mL of the extracts (*p* < 0.05). Conversely, a significant reduction in fluorescence (*p* < 0.01) was observed for the *S. pratensis* extract at all the incubation times when tested at 1 mg/mL compared with 0.5 mg/mL (36.44 ± 0.17%, 33.3 ± 0.70%, and 27.15 ± 2.59% vs. 61.25 ± 1.23%, 58.38 ± 0.14%, and 61.74 ± 1.75% at 1, 4, and 7 days, respectively) probably due to the prevalence of inhibitory effects at the highest tested concentration rather than synergistic effects among the different metabolites constituting the extract. Generally, after 4 days of incubation, all the tested extracts still maintain good antiglycative properties, especially *R. canina*, which reached 66.09 ± 1.29% of inhibitory activity. This is in contrast to *A. abrotanum*, which was completely inactive. A similar trend was also observed after 7 days of incubation. At each monitoring time, AG was always significantly more active than the extracts (*p* < 0.05), even at 1 mg/mL. Interestingly, *V. thapsus* had a near constant activity at all the incubation times both when tested at 0.5 mg/mL and 1 mg/mL (Figure 2).

All the tested extracts differently inhibited AGE formation. *R. canina* was the most active, followed by *C. sativa*, *A. abrotanum*, *B. umbellatus*, *V. thapsus*, *S. pratensis*, *S. officinalis*, and *A. officinalis*, which had activity values ranging from about 10 to 57% depending on concentration and monitoring time. Conversely, *B. vulgaris* was the least active in all the experimental conditions. The behavior of this extract could be related to general AGE formation, which is a complex process. In fact, it results from several reversible chemical reactions that simultaneously occur in the same system where they are formed and where the AGE α-dicarbonyl intermediate precursors are reorganized. For this reason, a linear trend during the monitoring time is not always found [33]. In addition, the antiglycative properties of plant species can be attributed to the different secondary metabolites present in the tested extracts, such as polyphenols, which are known to inhibit AGE formation through different mechanisms of action. In fact, these properties are often correlated with the ability of phenolic compounds to reduce oxidative stress, which is a key factor in the glycation process; to trap dicarbonyl compounds; or to directly interfere with the glycation reaction by amino group binding, as reported in the literature in the last decade [34]. For example, the antiglycative properties of some secondary metabolites, such as syringic acid, rutin, resveratrol, chlorogenic acid, or catechins, prevented AGE formation by reducing free radical formation, trapping MGO, or protecting potential protein binding sites [35]. The phenolic acid and flavonoid structures differently contribute to the antioxidant properties and trapping capacity of dicarbonyl compounds according to the number and position of hydroxyl group substituents [36]. In fact, luteolin with hydroxyl groups in the C3, C4, C5, and C7 positions inhibited the glycation process more efficiently than naringenin, which lacks the hydroxyl group in C3, thus highlighting the possible involvement of the C3 position in AGE inhibition [37]. For example, *Flos Sophorae Immaturus* and *Radix Scutellariae* extracts, characterized by a high flavonoid content, were able to totally inhibit AGE formation [38]. Similarly, ethyl acetate and butanolic fractions obtained from *Annona muricata* leaves effectively inhibited AGE formation, showing low IC50 values (166.1 ± 21.6 and 413.2 ± 49.5 μg/mL, respectively), and the activity has been ascribed to the composition of their secondary metabolites (quercetin-di-glucoside, quercetin-glucosyl-pentoside, rutin, chlorogenic acid, procyanidin B2 and C1, and (epi)catechin) [39]. Differently, ellagic, caffeic, gallic, and syringic acids stabilize the BSA structure and prevent AGE formation through interactions with lysine and arginine residues in the BSA active site. In particular, gallic acid has a low affinity for BSA due to the high number of hydroxyl groups, which reduce the interactions with the hydrophobic protein portion [40].

**Figure 3:**
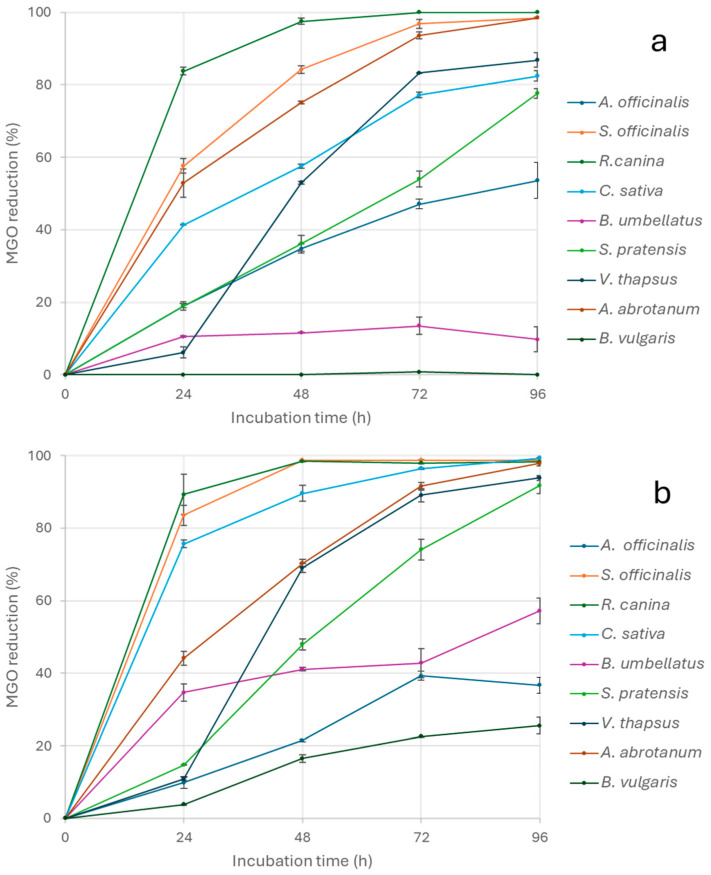
MGO trapping capacity of the extracts tested at 0.5 mg/mL (**a**) and 1 mg/mL (**b**).

In order to deeply investigate the antiglycative properties of the tested extracts, the capacity to directly trap MGO and GO (important promoters of the glycation process) was also evaluated over time by incubating each extract (0.5 and 1 mg/mL) with the α-dicarbonyl compounds for 24, 48, 72, and 96 h [41]. All extracts efficiently trapped MGO, and most of them linearly increased the activity over time with the exception of *B. vulgaris*, for which no reduction in MGO concentration was observed when tested at 0.5 mg/mL (Figure 3a) and only a slight reduction (25.54 ± 6.37%) was registered after 96 h when tested at 1 mg/mL (Figure 3b). Generally, a dose-dependent MGO trapping capacity was observed, except for *A. officinalis* and *A. abrotanum*. However, for this last extract, the trapping activity was similar at both concentrations after 96 h of incubation. *R. canina* was able to trap over 80% MGO after incubation for 24 h, reaching about 100% after incubation for 48 h, regardless of the tested concentration (Figure 3a,b). In addition, 1 mg/mL *S. officinalis* was able to almost completely trap MGO starting from 48 h of incubation of the system, and its activity was constant until the end of the monitoring time (Figure 3b).

**Figure 4:**
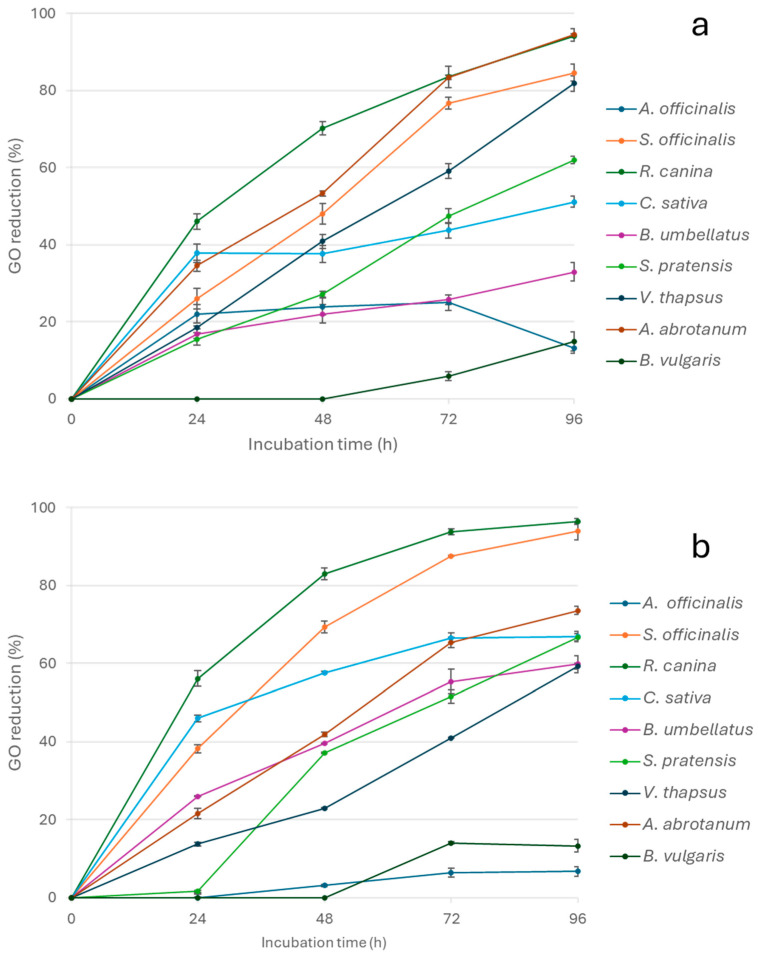
GO trapping capacity of the extracts tested at 0.5 mg/mL (**a**) and 1 mg/mL (**b**).

Generally, all extracts had higher trapping capacity against MGO than GO, even if the kinetic trends were similar. *B. vulgaris* confirmed its poor activity, trapping less than 20% of GO after 96 h of incubation at both concentrations tested. Similarly, the trapping capacity of *A. officinalis* was overall no higher than 6% at 1 mg/mL, and the extract was able to trap no more than 25% of GO after 72 h of incubation at 0.5 mg/mL. In addition, for *A. abrotanum* a higher GO trapping capacity was registered at the lowest concentration, thus indicating the absence of a dose-response activity toward GO for *A. officinalis* (Figure 4a,b). Differently, *A. abrotanum* reduced GO by 94.41 ± 1.55% and 73.56 ± 1.07% after 96 h at 0.5 mg and 1 mg/mL, respectively. All the other extracts showed a dose-dependent trapping capacity, consistently reaching activity values higher than 84% for *S. officinalis* and *R. canina* at the end of the monitoring time (Figure 4b).

The obtained results highlighting the higher trapping capacity against MGO than GO confirmed the data reported in the literature for other plant extracts, and it could be correlated to the different polyphenolic compositions. In fact, recent studies performed by Li et al. [42] indicated that quercetin was able to trap MGO more rapidly than GO. It has been suggested that MGO could form mono- or di-adducts. This is in contrast to GO, which is mainly present in the hydrated monomeric form in aqueous solution. The hydrated monomeric form easily tends to polymerize into dimeric or trimeric structures, which are in equilibrium with each other. Therefore, the GO trapping efficiency was lower than that of MGO due to lower concentrations of GO in the free form. Similar results were also obtained for epigallocatechin-3-gallate [43], luteolin, apigenin, hesperetin [44], and myricetin [45]. The flavonoid kinetics and trapping efficiency are also strongly affected by the number and position of hydroxyl groups as well as the presence of double bounds. In fact, apigenin had lower trapping capacity than naringenin due to the double bond in the C2-C3 positions on the C ring. Differently, the hydroxyl groups in C3 and C5 of kaempferol positively affected trapping capacities [46].

**Figure 5:**
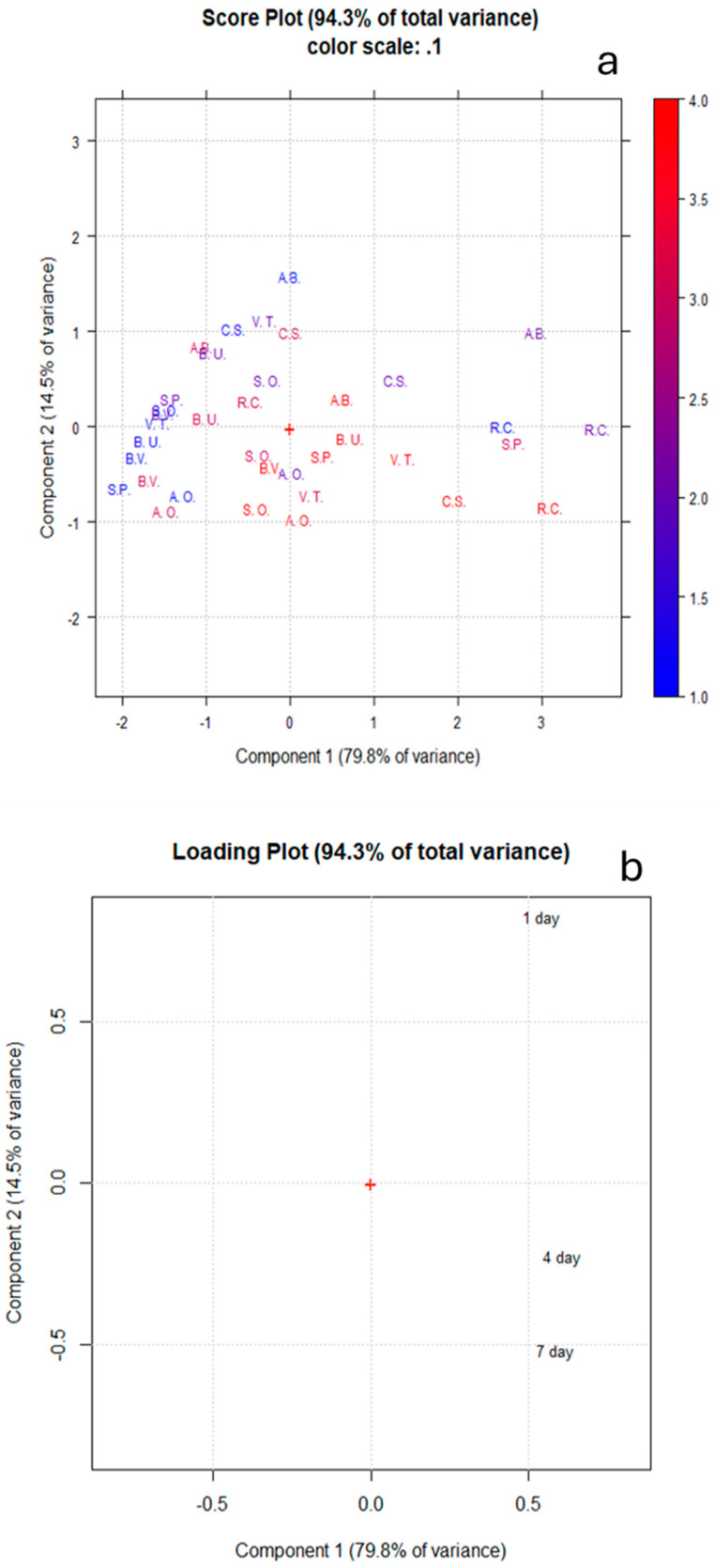
PCA of BSA-MGO assay data. (**a**) Score plot. Each acronym is related to a specific plant species (A.B. = *A. abrotanum*, B.U. = *B. umbellatus*, B.V. = *B. vulgaris*, C.S. = *C. sativa*, S.O. = *S. officinalis*, R.C. = *R. canina*, V.T. = *V. thapsus*, S.P. = *S. pratensis*, A.O. = *A. officinalis*). The color scale corresponds to the following data: 1 = inhibition of argpyrimidine-like formation at 0.5 mg/mL, 2 = inhibition of argpyrimidine-like formation at 1 mg/mL, 3 = inhibition of vesperlysine-like formation at 0.5 mg/mL, 4 = inhibition of vesperlysine-like formation at 1 mg/mL. (**b**) Loading plot.

To reduce the dimensionality of the antiglycative data and summarize the differences between the samples, principal component analysis (PCA) was performed. In particular, by applying PCA, new principal components (PCs), which are independent from the original variables (vesperlysine-like and argpyrimidine-like AGE inhibitory activity values), were generated. The graph of the score (displaying the position of each extract sample in the new coordinate space) and loading (displaying the relative contributions of each variable in calculating the scores) plots of the plant extract bioactivity data are shown in Figure 5a,b. The PC1 and PC2 described 94.30% of the total variance in the model, explaining 79.80% and 14.50% of the variability in the original observations, respectively. In particular, the data in the score plot (Figure 5a) were distributed mainly along PC1 from left to right following the activity increases. In the loading plot (Figure 5b), it was evident that the variables 4 days and 7 days, which are near to each other, are positively correlated, showing the same contribution. This is in contrast to the variable 1 day, which is separated from the other variables, showing a different contribution. However, they are all far from zero, thus showing a strong influence on the components. Thus, a different capacity of the extracts to inhibit AGEs at the two concentrations tested and a greater activity toward vesperlysine-like AGEs was evident. The distribution of the data in the score plot confirmed the different behaviors of the tested plant extracts, showing most clearly the higher activity of *R. canina*, especially in inhibiting the formation of argpyrimidine-like AGEs at both concentrations, but also the formation of vesperlysine-like AGEs at 1 mg/mL. *A. abrotanum* efficiently inhibited argpyrimidine-like AGE formation at 1 mg/mL, and *C. sativa* efficiently reduced the production of vesperlysine-like AGEs at 1 mg/mL. Finally, from the score plot, it was evident that *S. pratensis* was the most active at the lowest concentration in inhibiting the generation of vesperlysine-like AGEs.

**Figure 6:**
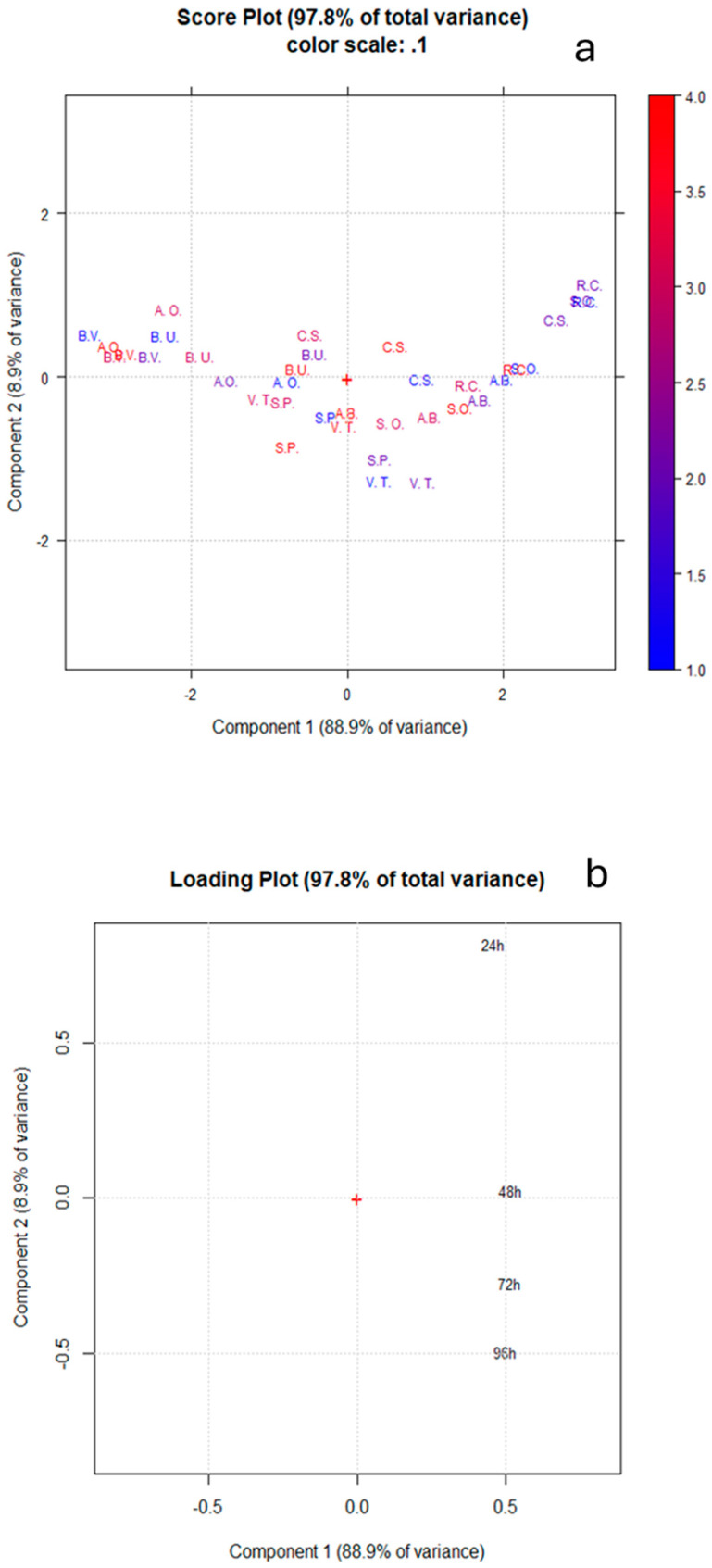
PCA of trapping MGO/GO assay data. (**a**) Score plot. Each acronym is related to a specific plant species (A.B. = *A. abrotanum*, B.U. = *B. umbellatus*, B.V. = *B. vulgaris*, C.S. = *C. sativa*, S.O. = *S. officinalis*, R.C. = *R. canina*, V.T. = *V. thapsus*, S.P. = *S. pratensis*, A.O. = *A. officinalis*). The color scale corresponds to the following data: 1 = trapping MGO at 0.5 mg/mL, 2 = trapping MGO at 1 mg/mL, 3 = trapping GO at 0.5 mg/mL, 4 = trapping GO at 1 mg/mL. (**b**) Loading plot.

In Figure 6a,b, the score plot and the loading plot of the PCA performed on the data obtained from trapping MGO/GO are shown, respectively. In this case, the observations were all plant extracts tested at two different concentrations, while the trapping MGO/GO activities at 24, 48, 72, and 96 h were the variables. PC1 and PC2 described 97.80% of the total variance in the model, explaining 88.90% and 8.90% of the variability in the original observations, respectively. Similarly to the previous score plot, the data were distributed mainly along PC1 from left to right, indicating that the activity increased (Figure 6a). The loading plot (Figure 6b) highlighted a correlation between the incubation times at 48, 72, and 96 h, which were close to each other, but separated from the first incubation time (24 h), indicating a different effect on the components that was significant in both cases (considering the distance from zero). The score plot confirmed a higher trapping capacity especially between 48 and 72 h of incubation at 1 mg/mL and a common higher activity toward MGO. In the plot *R. canina*, *S. officinalis*, *C. sativa*, and *A. abrotanum* were more separated from the other species, being the most active in trapping MGO. In contrast, *B. vulgaris* was the least active toward both MGO and GO, at both the tested concentrations.

**Figure 7:**
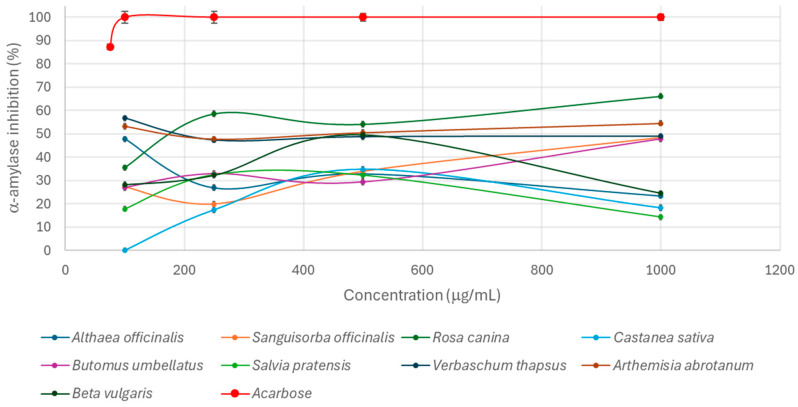
Hypoglycemic activity of the tested plant extracts (100–1000 µg/mL); acarbose was used as positive control (100–1000 µg/mL).

Considering that hyperglycemia could induce AGE formation, another possible mechanism of the extracts to reduce the glycation process could be α-amylase inhibition. Therefore, an in vitro assay was set up to evaluate the extracts’ capacity to inhibit α-amylase activity. α-Amylase is an enzyme mainly involved in the metabolism of polysaccharides at the intestinal level; therefore, its inhibition may control blood glucose levels by reducing the digestion and absorption of carbohydrates in the gastrointestinal tract [47]. Each extract was tested at different concentrations in the range of 0.1–1 mg/mL to evaluate dose-response activity, and the results were compared to acarbose, a well-known hypoglycemic agent that was used as a positive control (tested in the range of 100–1000 μg/mL). All extracts inhibited α-amylase, reducing starch hydrolysis by 15–65% at the highest tested concentration. However, for only a limited number of extracts, it was possible to detect dose-response activity starting from 250 μg/mL (Figure 7).

The activity registered for the extracts at each concentration was significantly lower than that registered for acarbose (*p* < 0.01) which had dose-dependent hypoglycemic activity in the range of 100–150 μg/mL and maintained the capacity to totally inhibit the enzymatic activity at higher concentrations, as also reported by Abu Soud et al. [48]. Considering the tested plant extracts, *V. thapsus* and *A. abrotanum* inhibited α-amylase with activity values higher than 40% at each concentration tested. This is in contrast to *S. pratensis* and *C. sativa*, which reduced α-amylase activity less than 40% despite increasing the concentration. *B. umbellatus*, *S. officinalis*, and *R. canina* inhibited the enzyme activity more than 45% (47.82 ± 0.89%, 48.44 ± 1.88%, and 66.20 ± 0.88%, respectively) at 1 mg/mL, while *B. vulgaris* reached the highest activity at 0.5 mg/mL (49.65 ± 1.65%). *V. thapsus*, *B. umbellatus*, and *S. officinalis* at 1 mg/mL were able to inhibit the α-amylase activity with similar values that were significantly higher than that registered for *B. vulgaris*, *A. officinalis*, *C. sativa*, and *S. pratensis* and lower than that for *R. canina* and *A. abrotanum* (*p* < 0.01). Overall, *R. canina* could be again identified as the most potential hypoglycemic extract, confirming its putative capacity to counteract the glycation reaction.

In addition, *C. sativa* was the only plant species completely inactive when tested at the lowest concentration (0.1 mg/mL) (Figure 7). Generally, the hypoglycemic properties registered for the tested samples were similar to those reported in the literature for other edible plant extracts and those included on the Belfrit list, which can be considered potential tools for the hyperglycemia treatment. For example, *Cinnamomum zeylanicum*, *Artocarpus altilis*, *Piper betel*, and *Artocarpus heterophyllus* methanol extracts reduced α-amylase activity by 50% at concentrations of 130.55 μg/mL, 118.88 μg/mL, 84.63 μg/mL, and 70.58 μg/mL, respectively [49]. Similarly, *Adenanthera pavonina* leaf methanolic extract exhibited higher hypoglycemic properties (IC_50_ = 16.16 ± 2.23 μg/mL) [50]. The absence of a dose-response activity could be explained by the presence of different compounds in plant extracts, such as polyphenols. In fact, they can inhibit α-amylase activity by forming hydrogen bonds between hydroxyl groups and catalytic site residues (Asp197, Glu233, and Asp3000), preventing the interaction with the substrate, or by hydrophobic interaction between the aromatic rings and the hydrophobic residues of the enzyme (Trp59), thus altering the conformation of the active site. In addition, the interaction between secondary metabolites and α-amylase can also induce conformational changes, reducing enzymatic activity [51]. Therefore, the polyphenols’ chemical features play a crucial role in the extracts’ efficacy. The presence of a high number of hydroxyl groups increases the interaction with α-amylase through hydrogen bonds with the active site of the enzyme, differently from glycosylation, methylation, or methoxylation, which decrease the inhibitory activity [52]. Conversely, the presence of a double bond conjugated with the 4-carbonyl group promotes the hydrophobic interaction with the residue Trp59 at the active site of the enzyme [51]. In addition, polyphenolic compounds can act through a competitive or noncompetitive mechanism for the substrate (starch) [53]. In fact, kinetic studies performed on epigallocatechin gallate (EGCG) and epigallocatechin 3-*O*-(3-*O*-methyl) gallate (EGCG3″Me) present in Oolong tea highlighted how these compounds competitively and noncompetitively act, respectively [54]. Therefore, the presence of different α-amylase inhibitory mechanisms needs further investigation to better understand the potential synergistic or inhibitory effect of the polyphenols present in the extracts. In fact, the inhibitory activity could be related to the total phenolic content or to a specific class of compounds; therefore, a better chemical characterization of the extracts is needed to elucidate the contribution of the individual inhibitors, their chemical structure, and the interaction among them [55].

**Figure 8:**
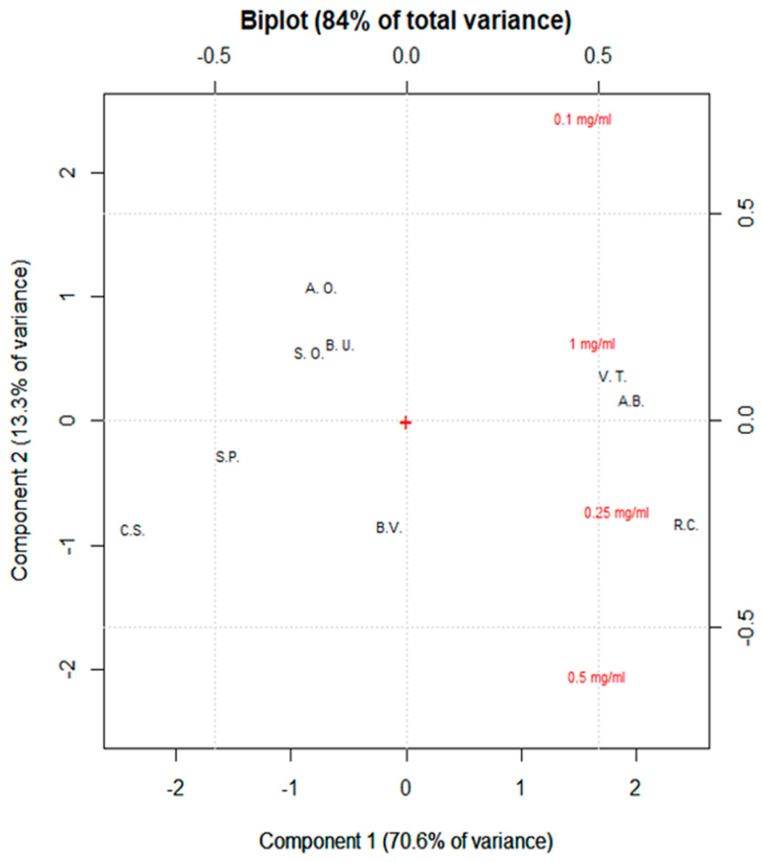
PCA of α-amylase assay data. Biplot is reported. Each acronym is related to a specific plant species (A.B. = *A. abrotanum*, B.U. = *B. umbellatus*, B.V. = *B. vulgaris*, C.S. = *C. sativa*, S.O. = *S. officinalis*, R.C. = *R. canina*, V.T. = *V. thapsus*, S.P. = *S. pratensis*, A.O. = *A. officinalis*).

Figure 8 shows the biplot of the PCA performed on the data obtained regarding the hypoglycemic properties. All plant extracts represent the observations, while the bioactivity registered at different concentrations are the variables. PC1 and PC2 explained 84.00% of total variance (PC1 70.60% and PC2 13.30%). *V. thapsus*, *R. canina*, and *A. abrotanum* were the species able to efficiently inhibit the α-amylase activity. *S. officinalis*, *B. umbellatus*, and *A. officinalis* had similar hypoglycemic properties (they are close to each other in the graph). This is in contrast to *S. pratensis*, *B. vulgaris*, and *C. sativa*, which are more separated from the other species. In addition, all variables showed a strong effect on the components, but with different contributions. Therefore, the biplot confirmed *R. canina* as the most active extract, even when tested at the lowest concentration, followed by *A. abrotanum* and *V. thapsus*. These extracts were followed by *S. officinalis*, *B. umbellatus*, *B. vulgaris*, and *A. officinalis*, which presented similar data distributions. Finally, *S. pratensis* and *C. sativa* were confirmed as the least active extracts.

**Figure 9:**
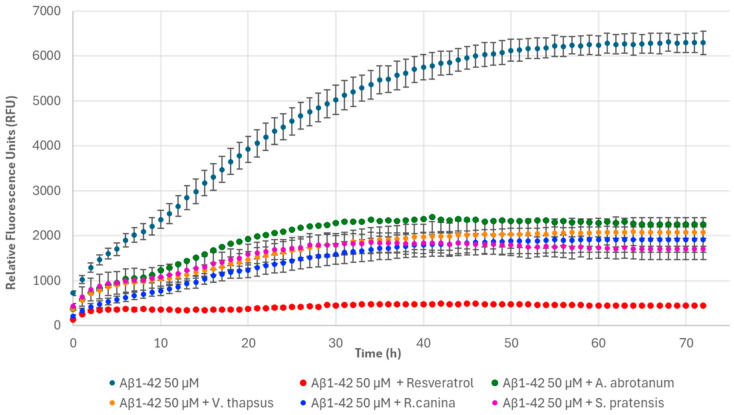
Fibril formation monitored over time based on ThT fluorescence of 50 μM Aβ1-42 alone, 100 μg/mL resveratrol, and in the presence of 1 mg/mL *A. abrotanum*, *V. thapsus*, *R. canina*, and *S. pratensis*.

**Figure 10:**
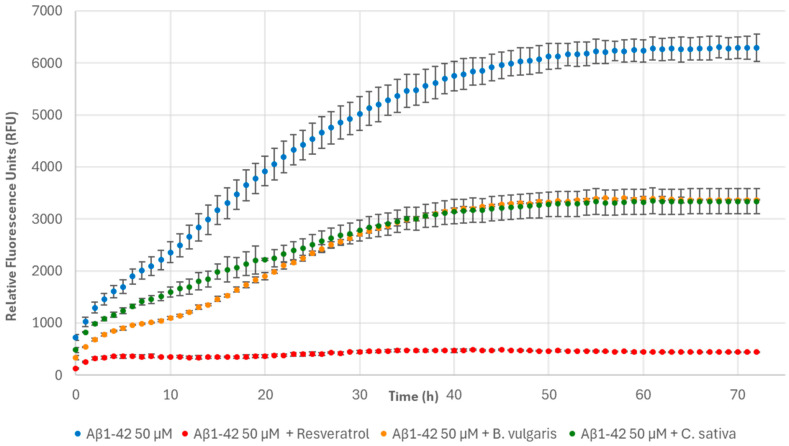
Fibril formation monitored over time based on ThT fluorescence of 50 μM Aβ1-42 alone, 100 μg/mL resveratrol, and in the presence of 1 mg/mL *B. vulgaris* and *C. sativa*.

**Figure 11:**
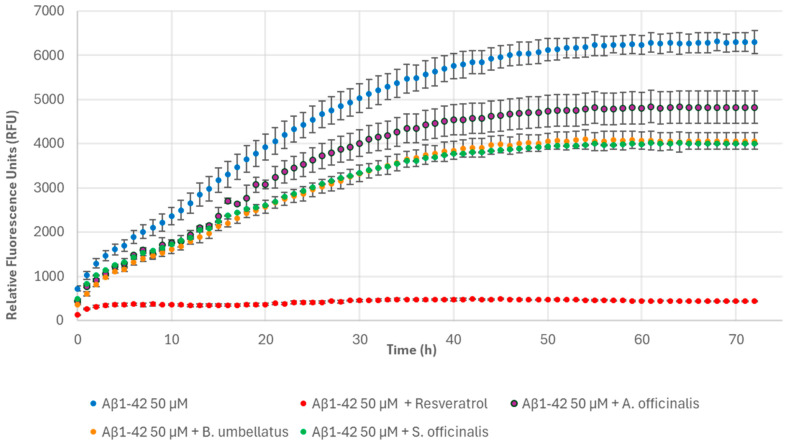
Fibril formation monitored over time based on ThT fluorescence of 50 μM Aβ1-42 alone, 100 μg/mL resveratrol, and in the presence of 1 mg/mL *A. officinalis*, *B. umbellatus*, and *S. officinalis*.

**Figure 12:**
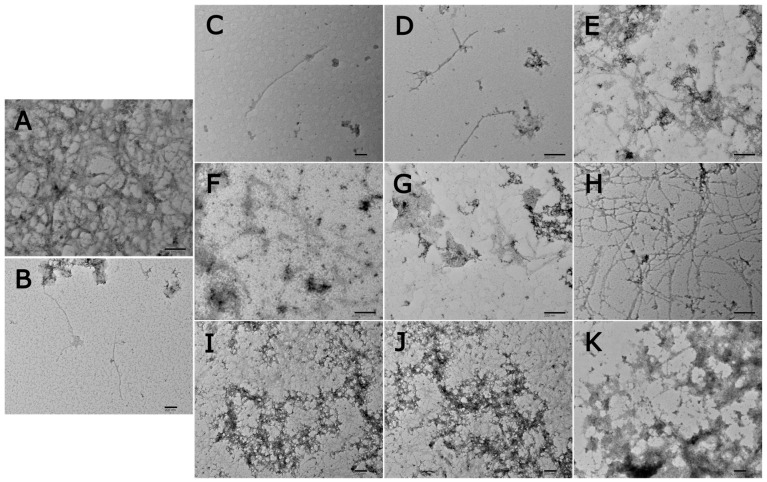
TEM images after 72 h of incubation with either 50 μM Aβ1-42 alone (**A**) or in the presence of 100 μg/mL resveratrol (**B**), 1 mg/mL *A. abrotanum* (**C**), *V. thapsus* (**D**), *R. canina* (**E**), *S. pratensis* (**F**), *B. vulgaris* (**G**), *C. sativa* (**H**), *A. officinalis* (**I**), *B. umbellatus* (**J**), and *S. officinalis* (**K**). Scale bar = 200 nm.

Considering that AGE formation is strictly related to the development of degenerative diseases, in the last step of the work, we investigated the ability of the extracts to inhibit the formation of Aβ1-42 fibrils using the ThT-fluorescence assay and TEM analysis. Aβ1-42 and Aβ1-40 are the two main component of amyloid plaques; therefore, they can be considered important pathological markers of AD. ThT is a fluorescent dye widely used to monitor the formation of Aβ1-42 amyloid fibrils in vitro due to its ability to bind amyloid fibrils, inducing the formation of a highly fluorescent complex. The complex formation is a consequence of the reduced rotational movement of the benzothiazole and aminobenzoyl rings of bound ThT, which results in increased fluorescence [56]. TEM is a useful technique for assessing the in vitro presence of amyloid fibrils or amorphous aggregates. The evaluation of the extracts’ anti-amyloidogenic properties first required the set-up of the fibrillogenesis process of Aβ1-42. The typical self-assembly process characterized by a sigmoidal growth was obtained [57], and it reached a plateau after 72 h, when mature amyloid fibrils were formed (Figure 9). Conversely, the relative fluorescence intensity of 50 μM Aβ1-42 incubated with resveratrol (100 μg/mL, used as a positive control) was reduced by 92.00 ± 0.01% after 72 h (Figure 9, Figure 10 and Figure 11), indicating a significant reduction of β-sheet fibril formation. In fact, incubation of Aβ1-42 peptide alone for 72 h at 37 °C generated a dense meshwork of mature amyloid fibrils, morphologically long and thick with few branches, as evident from the TEM results (Figure 12A). TEM analysis confirmed the presence of a few mature amyloid fibrils and the prevalence of soluble oligomeric forms (Figure 12B). These results were in agreement with the literature data, according to which resveratrol was able to inhibit Aβ1-42 fibrillation but not oligomerization [58].

Next, the ability of extracts to inhibit the fibrillogenesis process was investigated. Each sample (1 mg/mL) was incubated with 50 μM Aβ1-42 for 72 h at 37 °C. In presence of *A. abrotanum*, *V. thapsus*, *R. canina*, and *S. pratensis*, reductions in relative fluorescence intensity of 64.09 ± 0.20%, 66.31 ± 5.04%, 69.05 ± 4.49%, and 72.21 ± 3.73% were observed, respectively (Figure 9). This trend was related to the reduced formation of amyloid fibrils. This is also evident in TEM images, which confirmed a significant decrease in the number of long and unbranched fibrils and the absence of a dense meshwork of mature amyloid fibrils, typical of free Aβ1-42 peptide. Moreover, the incubation of Aβ1-42 with *A. abrotanum* (Figure 12C), *V. thapsus* (Figure 12D), and *S. pratensis* (Figure 12F) for 72 h induced a morphological change in the fibrils, indicating an effect of the extracts on the shape of the Aβ1-42 fibrils. In particular, the addition of *A. abrotanum* and *V. thapsus* produced few fibrils of intermediate length, preventing their elongation process and stabilizing the protofibrils. Differently, a mixture of intermediate fibrils and short fragments was obtained when *S. pratensis* is present in the reaction mixture. Only the incubation of Aβ1-42 with *R. canina* (Figure 12E) produced very few long and thick fibrils, morphologically similar to those generated by Aβ1-42, although in a significantly reduced amount. Therefore, the presence of *R. canina* had a low influence on the shape of the Aβ1-42 fibrils.

The incubation of Aβ1-42 with *B. vulgaris* and *C. sativa* had less of an effect and produced 46.04 ± 0.16% and 46.45 ± 3.86% reductions in fibril formation, respectively, compared to the control (Aβ1-42) (Figure 10). In addition, these samples mainly differed from Aβ1-42 in the density of the fibrillar meshwork. In fact, *C. sativa* induced the formation of a fibrillar meshwork characterized by long and thick mature amyloid fibrils, morphologically similar to those formed by the control, but with a lower fibril density (Figure 12H). A reduced fibrillar density was also evident in the presence of *B. vulgaris.* In this case, a lower population of long and thick fibrils, and many intermediate-length and thinner fibrils were present (Figure 12G).

Finally, no morphological change and no change in fibrillar density were observed following the incubation of Aβ1-42 with *A. officinalis*, *B. umbellatus*, and *S. officinalis*. In fact, the relative fluorescence intensity indicated a poor effect on the fibrillogenesis process because it was reduced by only 23.03 ± 5.77%, 35.32 ± 2.96%, and 36.26 ± 1.20%, respectively, compared to the control (Figure 11). In addition, the incubation of Aβ1-42 with the extracts produced a dense fibrillar meshwork of fibrils that were morphologically similar in diameter and length to those produced by Aβ1-42 peptide alone (Figure 12I–K).

Therefore, the obtained results indicated that the most active plant extracts (*A. abrotanum*, *S. pratensis*, *R. canina*, and *V. thapsus*) were able to inhibit the fibrillogenesis process, reducing the formation of fibrils, interfering with the fibril maturation process, and also affecting their shape. This behavior was previously reported by Kumar et al. [59] for the aqueous extract of *W. somnifera* L. *Dunal* roots, which inhibited in vitro Aβ1-42 fibrillogenesis, reducing the formation of mature amyloid fibrils and promoting the formation of shorter fibrils than the control. The main effect observed was a reduction in the density of the fibrillar meshwork, as noted for *B. vulgaris* and *C. sativa*, down to an almost total absence of an effect on the fibrillogenic process, as observed in the presence of *A. officinalis*, *S. officinalis*, and *B. umbellatus*. It is reasonable to attribute these different anti-amyloidogenic properties to the various compositions of the secondary metabolites of the tested plant extracts, which are known in the literature to inhibit the aggregation of Aβ amyloid peptides, as in the case of quercetin, rosmarinic acid, luteolin, gallic acid, EGCG, and kaempferol [60,61]. Plant secondary metabolites could interfere with the different stages of the fibrillogenesis process, starting from the inhibition of native to misfolded conformational changes, inhibiting the production of oligomeric species or the polymerization phase, or favoring the disaggregation of preformed aggregates. For example, myricetin forms hydrophobic interactions with oligomeric forms, preventing their aggregation, and inhibits β-sheet conformational changes and the fibrils’ elongation process. Differently, tannic acid inhibits Aβ1-40 and Aβ1-42 fibrillation and extension and destabilizes preformed Aβ fibrils [62].

A behavior similar to *A. abrotanum* and *V. thapsus* was previously highlighted for *Allium roseum* L. extract. Kaempferol was identified as the most abundant compound, and TEM images obtained from the incubation of Aβ1-42 with the extract and with kaempferol alone showed the absence of fibrils and the presence mainly of amorphous aggregates [63]. Similarly, EGCG, epicatechin 3-*O*-gallate, and catechin 3-*O*-gallate isolated from fermented tea (*Camellia sinensis*) inhibited fibrillogenesis processes by 78%, 62.8%, and 46.4%, respectively, promoting the formation of oligomers and linear tangled Aβ1-42 fibrils [64], and similar effects were noted for many tested extracts. In addition, gallic acid inhibited the formation of mature Aβ1-40 amyloid fibrils according to Liu et al. [65]. The anti-fibrillogenic properties of gallic acid were also confirmed by Andrade et al. [66], according to which the incubation of Aβ1-42 with gallic acid promoted the formation of short fragments, preventing the formation of Aβ1-42 fibrils, with a mechanism similar to that reported for *S. pratensis*. Differently, quercetin reduced the process of Aβ1-40 fibrillogenesis by 85% by interacting with Aβ1-40 in the early stages of nucleation, altering the normal process of fibril formation and promoting the formation of nonamyloid aggregates [67].

## 4. Conclusions

In this work, the anti-glycative, hypoglycemic, and anti-fibrillogenic properties of nine edible plant extracts were investigated, highlighting their in vitro effects on the factors that have a great impact on metabolic and age-related diseases. In general, all extracts were able to counteract AGE formation, especially at the intermediate stage of the glycation reaction. In addition, they had great MGO and GO trapping capacity and promising hypoglycemic properties. In particular, *R. canina* was the most active species given its anti-glycative, hypoglycemic, and anti-fibrillogenic activities.

Although these properties could be ascribed to the secondary metabolites (mainly polyphenols considering the solvent used to generate the extract) present in the edible extracts, metabolic profile characterization is mandatory to better define the correlation between the extract composition and the biological properties as well as the potential mechanism of action. The data discussed in the present work are preliminary, and the next step of the research will include bioaccessibility/bioavailability, stability, and safety investigations, after a complete chemical characterization of the extract/s. Using this approach, it will be possible to obtain useful food ingredients that also should be tested in vivo, since it is known that the biological activity obtained under in vitro and in vivo conditions could be different.

In conclusion, the studies reported here highlight new potential sources of bioactive compounds that can contribute to the development of functional products/ingredients with health benefits. These findings also exploit the rich Italian biodiversity and open a range of possibilities for economic development and public policies.

## Figures and Tables

**Table 1:** List of collected plant species with their sampling features. *n* = number of plants from which the plant material was collected and pooled.

Species	Family	Place of Sampling/Supplier	*n*	Organs Collected
*Artemisia abrotanum* L.	Asteraceae	Padua Botanical Garden	2	Leaves and young stems
*Salvia pratensis* L.	Lamiaceae	Veneto Agricoltura nursery *	2	Leaves
*Althaea officinalis* L.	Malvaceae	Veneto Agricoltura nursery *	2	Leaves
*Butomus umbellatus* L.	Butomaceae	Padua Botanical Garden	5	Leaves and young stems
*Castanea sativa* Mill.	Fagaceae	Padua Botanical Garden	1	Leaves
*Verbascum thapsus* L.	Scrophulariaceae	Padua Botanical Garden	2	Leaves
*Sanguisorba officinalis* L.	Rosaceae	Padua Botanical Garden	5	Leaves and young stems
*Rosa canina* L.	Rosaceae	Padua Botanical Garden	2	Leaves
*Beta vulgaris* L.	Amaranthaceae	University of Verona	10	Leaves

* Centre for Biodiversity and Outside Forest Activities (Montecchio Precalcino, Vicenza, Italy).

## Data Availability

The original contributions presented in this study are included in the article. Further inquiries can be directed to the corresponding author.
